# Rituximab in the Treatment of Acquired Angioedema Secondary to Marginal Zone Lymphoma of the Spleen

**DOI:** 10.7759/cureus.36790

**Published:** 2023-03-28

**Authors:** Kathie Wu, Joseph Vadakara

**Affiliations:** 1 Internal Medicine, Geisinger Medical Center, Danville, USA; 2 Hematology/Oncology, Geisinger Medical Center, Danville, USA

**Keywords:** spleen, marginal zone lymphoma, lymphoma, angioedema, rituximab

## Abstract

Angioedema occurs in less than 1-2% of the population and amongst these cases, those with acquired angioedema are less prevalent than hereditary angioedema. Amongst cases of acquired angioedema, studies have shown that they were highly linked with an associated lymphoproliferative disorder, suspected secondary to the production of neutralizing autoantibodies from pathological B cell proliferation. We present a case of a patient who presented with recurrent episodes of angioedema and was found to have low C4 and C1 esterase function, initially concerning for a hereditary angioedema variant, who was subsequently found to have marginal B cell lymphoma mimicking hereditary angioedema.

## Introduction

Acquired angioedema (AAE) occurs in approximately 1 in every 100,000 to 500,000 people due to an acquired deficiency of the inhibitor of the first component of complement (C1-INH) [1]. This leads to clinical features of recurrent localized swellings of the face, upper respiratory tract, and gastrointestinal tract. While rare, acquired angioedema has been closely associated with lymphoma as well as other lymphoproliferative disorders including monoclonal gammopathy of uncertain significance (MGUS). An estimated 33% of patients with acquired angioedema have or will develop non-Hodgkin’s lymphoma. Hereditary angioedema, however, resulting from a mutation in the SERPING1 gene for C1 inhibitor protein, presents with the same clinical symptoms as acquired angioedema, but has an earlier age of symptom onset, often by ages five to eleven years old [2,3]. We describe a case of acute acquired angioedema that ultimately led to the diagnosis of marginal zone lymphoma of the spleen.

## Case presentation

An 86-year-old female with no significant past medical history presented to the hospital with concerns of lip and facial swelling that did not relieve with diphenhydramine at home. On admission, she was given solumedrol, famotidine, and intravenous diphenhydramine with no improvement in her symptoms. She began to develop voice changes concerning for airway compromise and was subsequently intubated for airway protection. The patient took no medications and had no prior history of angioedema or similar swelling in the past. She was treated with dexamethasone therapy with improvement in the swelling and was successfully extubated. Initial testing through immunology revealed low C4 and C1 esterase function concerning for a hereditary angioedema variant. The patient had no history of autoimmune disease, vasculitis, nephropathy, or liver disease that could provide an alternative explanation for her hypocomplementemia. Repeat C1 esterase function testing and C4 levels were sent to confirm the diagnosis.

**Figure 1:**
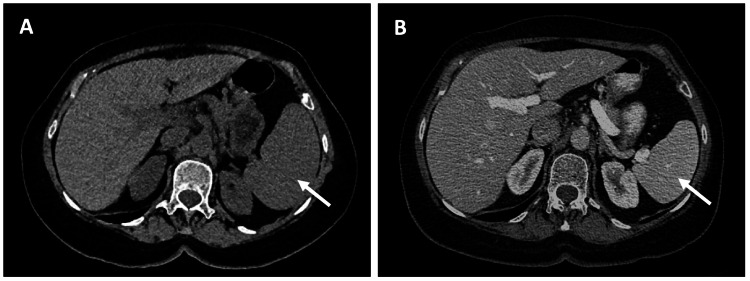
(A) Massive splenomegaly. (B) Reduction of splenomegaly post rituximab therapy.

**Figure 2:**
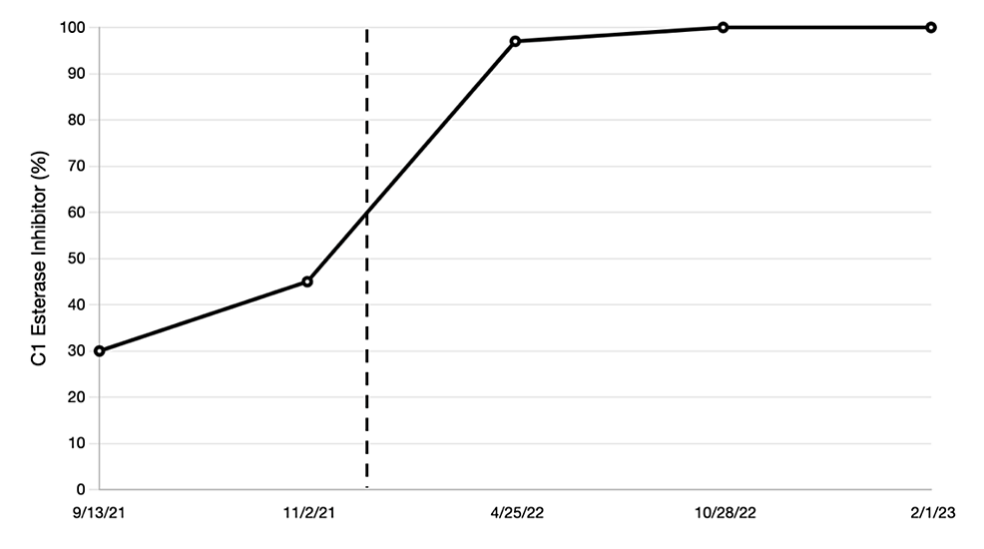
Line graph demonstrating the trend of functional C1 esterase inhibitor levels. The dashed line represents the initiation of rituximab therapy.

**Figure 3:**
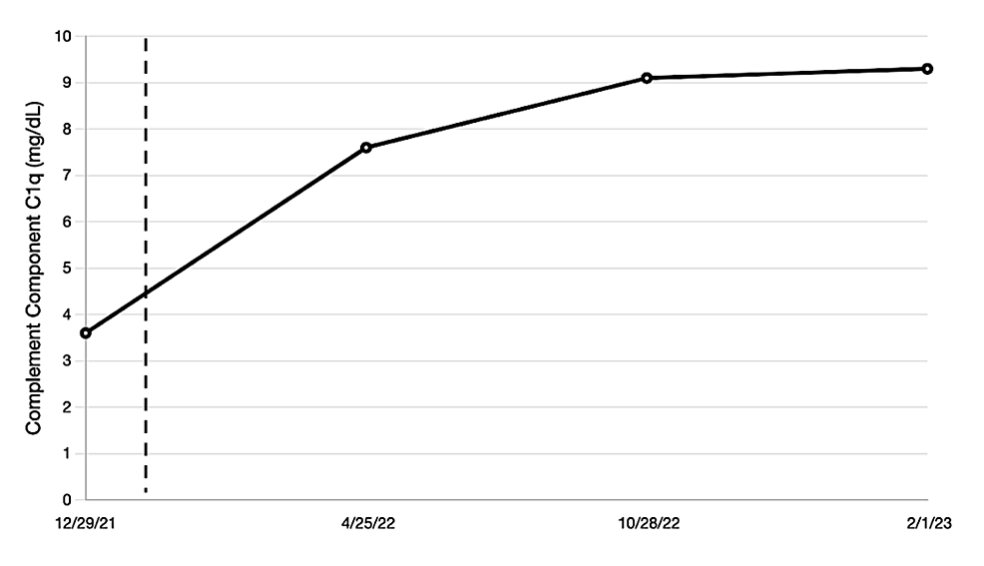
Line graph demonstrating the trend of complement component C1q levels. The dashed line represents the initiation of rituximab therapy.

A month later, the patient had another episode of lip and tongue swelling with associated shortness of breath requiring intubation. Due to the suspicion for angioedema, she was treated with Berinert which improved her symptoms. Her repeat blood work redemonstrated low levels of C1 esterase and complement concerning for hereditary angioedema. Given that the onset of age for hereditary angioedema commonly occurs in younger populations, there was high suspicion that the patient had an acquired form of angioedema mimicking hereditary angioedema. Further workup for malignancy was performed with CT imaging revealing massive splenomegaly (Figure 1A) and serum protein electrophoresis showing elevation in IgG kappa-free light chains concerning for myeloma. Flow cytometry analysis revealed small monotypic B cells which in conjunction with the splenomegaly, was concerning for a marginal zone lymphoma of the spleen. Treatment was initiated with weekly rituximab for four weeks and repeat imaging a few months later showed significant improvement in splenomegaly (Figure 1B). Blood work monitored during the course of treatment also demonstrated improvement of both functional C1 esterase inhibitor (Figure 2) and complement component C1q levels (Figure 3) post-rituximab therapy. The patient has not had any further flares of angioedema for the last 14 months. 

## Discussion

Acquired and hereditary angioedema share similar clinical features but differ in the typical age of onset with acquired angioedema occurring typically after the age of 40 [3]. Symptoms include recurrent swelling of the upper respiratory tract and the gastrointestinal tract. Deficiency in C1 esterase inhibitor protein levels leads to a build-up of bradykinin and increased vascular permeability, resulting in angioedema. Studies have shown that as many as 33% of patients with acquired angioedema due to an acquired C1 inhibitor deficiency either has or will develop a non-Hodgkin lymphoma [4]. While the precise etiology of acquired C1 esterase inhibitor protein remains ill-defined, in B cell lymphomas, pathology has shown that low levels of C1 inhibitor are secondary to the production of anti-C1-inhibitor autoantibodies or due to consumption of inhibitor by pathological lymphatic tissues [4,5]. Therefore, the standard treatment for angioedema attacks involves replacement therapy with C1 inhibitor concentrate as our patient had. However, studies have shown that patients may become less responsive to inhibitor concentrate treatment due to the production of autoantibodies over time, thereby requiring treatment of the underlying disorder [1]. In one study looking at the follow-up of 72 patients with acquired C1 esterase inhibitor deficiency, 24 patients were diagnosed with lymphoma, eight of which were diagnosed with a low-grade nonfollicular B cell lymphoma at the time of onset of angioedema, with a median age of 57.5 at the onset of angioedema symptoms and diagnosis of lymphoma at 63 years. Of those 24 patients, eighteen were treated with chemotherapy while only two were treated with rituximab alone, with only patients treated with chemotherapy noting a reduction in angioedema symptoms [4]. In another published report, a 50-year-old male was diagnosed with non-Hodgkin lymphoma after presenting with angioedema and was again treated with chemotherapy with no further recurrence of angioedema [6]. Our patient was unique given her significantly later age onset of angioedema and diagnosis, which also drove the decision to forgo traditional chemotherapy due to concern for worsening toxicities and proceed with treatment with rituximab alone [7]. Despite the above literature showing the majority of patients being treated with chemotherapy, our patient was still able to achieve remission of angioedema flares with rituximab therapy alone. Her labs were also able to demonstrate normalization of C1 esterase inhibitor levels and C1q following the completion of rituximab.

## Conclusions

In this patient, her immunological workup could not differentiate between hereditary or acquired angioedema, but the late onset of her symptoms suggested a nonhereditary cause of her symptoms. This case highlights the importance of maintaining a high degree of suspicion for malignancy, particularly lymphoma, in patients with new, late-onset symptoms of angioedema. Furthermore, though chemotherapy may be considered first-line treatment for this population, particularly when the median ages of onset are 50-60 years, consideration should be given to alternative therapy as patients may still achieve remission of symptoms without the increased risks of chemotherapy toxicity in advanced age.
